# *QuickStats*: Prevalence* of Edentualism^^†^^ in Adults Aged ≥65 Years, by Age Group and Race/Hispanic Origin — National Health and Nutrition Examination Survey, 2011–2014

**DOI:** 10.15585/mmwr.mm6603a12

**Published:** 2017-01-27

**Figure:**
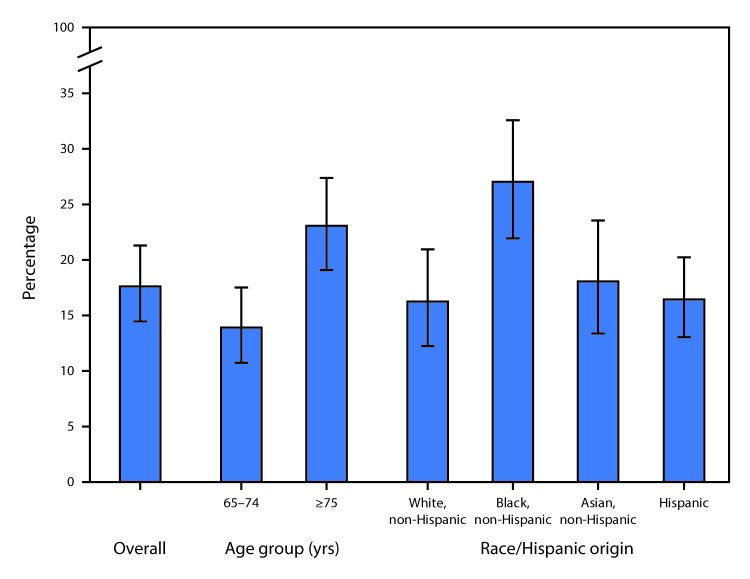
During 2011–2014, 17.6% of adults aged ≥65 years were edentulous or had lost all their natural, permanent teeth. Adults aged ≥75 years (23.0%) were more likely to be edentulous compared with adults aged 65–74 years (13.9%). Non-Hispanic black adults aged ≥65 years were more likely to be edentulous (27.0%) compared with non-Hispanic white (16.2%), non-Hispanic Asian (18.0%), and Hispanic adults (16.4%) aged ≥65 years.

**Source:** CDC/NCHS. National Health and Nutrition Examination Survey Data. Hyattsville, MD: US Department of Health and Human Services, CDC, National Center for Health Statistics; 2011–2014. https://www.cdc.gov/nchs/nhanes.htm.

